# Supplementary material for: A Systematic Review of Child Health and Developmental Outcomes Associated with Low Birthweight and/or Small for Gestational Age in Indigenous Children from Australia, Canada and New Zealand
Source: Int J Environ Res Public Health. 2021 Dec 1;18(23):12669. doi: 10.3390/ijerph182312669 (PMC8657270; doi:10.3390/ijerph182312669)
Supplement: Supplementary file 1 [file ijerph-18-12669-s001.zip › ijerph-1442850-supplementary.pdf]

Supplementary Material

Supplementary Table S1 – Search Terms

Pubmed Search Terms

#1 Title/Abstract

("Aborigin*" OR "Indigenous") AND ("Australia*" OR "New-Zealand*" OR "Canada*")

#2 Title/Abstract

"Anangu*" OR "Arrernte*" OR "Koori*" OR "Goorie*" OR "Tiwi*" OR "Pitjantjatjara*" OR "Luritja*" OR "Warlpiri*" OR "Torres-Strait-Islander*" OR "First-Nation*" OR "Inuit*" OR "Metis*" OR "Eskimo*" OR "Cree" OR "Crees" OR "Iroquois*" OR "Tlingit*" OR "Kainai*" OR "Algonquin*" OR "Anishinaabe*" OR "Dene" OR "Denes" OR "Nakoda*" OR "Syilx*" OR "Maori*" OR "Moriori*" OR "tangata-whenua*" OR "Ngapuhi*" OR "Ngati-Porou*" OR "Ngai-Tahu*" OR "Waikato*" OR "north-american-indian*"

#3 Title/Abstract

"Dental" OR "teeth" OR "gum" OR "enamel" OR "body-mass-index" OR "bmi" OR "respiratory" OR "asthma*" OR "disabilit*" OR "vision" OR "eye" OR "sight" OR "eyesight" OR "ophthalmic" OR "heart" OR "cardiac" OR "cardio*" OR "hear" OR "hearing" OR "deaf*" OR "disease*" OR "skin" OR "infection*" OR "development*" OR "milestone*" OR "behaviour*" OR "behavior*" OR "language" OR "speech" OR "learning" OR "academic" OR "anxiety" OR "depression" OR "suicid*" OR "health" OR "injur*" OR "illness*" OR "hospital*" OR "health-service*" OR "resilience" OR "strength*"

#4 Title/Abstract

"Low-birth-weight" OR "small-for-gestational-age" OR "intrauterine-growth-restrict*" OR "intrauterine-growth-retard*" OR "fetal-growth-restrict*" OR "foetal-growth-restrict*" OR "preterm" OR "pre-term" OR "prematur*"

#5 Title/Abstract

"newborn*" OR "new-born*" OR "baby" OR "babies" OR "neonat*" OR "neo-nat*" OR "infan*" OR "toddler*" OR "pre-schooler*" OR "preschooler*" OR "kinder" OR "kinders" OR "kindergarten*" OR "kinder-aged" OR "boy" OR "boys" OR "girl" OR "girls" OR "child" OR "children" OR "childhood" OR "pediatric*" OR "paediatric*" OR "school-age*" OR "schoolage*" OR "schoolchild*" OR "schoolgirl*" OR "schoolboy*" OR "adolescen*" OR "youth" OR "youths" OR "teen" OR "teens" OR "teenage*"

#6 **All fields**

NOTNLM OR publisher[sb] OR inprocess[sb] OR pubmednotmedline[sb] OR indatareview[sb] OR pubstatusaheadofprint

#7 (#1 OR #2) AND #3 AND #4 AND #5

Limits: English language; yr="2000 -Current"; and excludes: books & documents or case reports or comment or editorial or guideline or letter or practice guideline

Scopus Search Terms

#1 Title/Abstract/Keyword

("Aborigin*" OR "Indigenous") W/1 ("Australia*" OR "New-Zealand*" OR "Canada*")

#2 Title/Abstract/Keyword

"Anangu*" OR "Arrernte*" OR "Koori*" OR "Goorie*" OR "Tiwi*" OR "Pitjantjatjara*" OR "Luritja*" OR "Warlpiri*" OR "Torres-Strait-Islander*" OR "First-Nation*" OR "Inuit*" OR "Metis*" OR "Eskimo*" OR "Cree" OR "Crees" OR "Iroquois*" OR "Tlingit*" OR "Kainai*" OR "Algonquin*" OR "Anishinaabe*" OR "Dene" OR "Denes" OR "Nakoda*" OR "Syilx*" OR "Maori*" OR "Moriori*" OR "tangata-whenua*" OR "Ngapuhi*" OR "Ngati-Porou*" OR "Ngai-Tahu*" OR "Waikato*" OR "north-american-indian*"

#3 Title/Abstract/Keyword

"Dental" OR "teeth" OR "gum" OR "enamel" OR "body-mass-index" OR "bmi" OR "respiratory" OR "asthma*" OR "disabilit*" OR "vision" OR "eye" OR "sight" OR "eyesight" OR "ophthalmic" OR "heart" OR "cardiac" OR "cardio*" OR "hear" OR "hearing" OR "deaf*" OR "disease*" OR "skin" OR "infection*" OR "development*" OR "milestone*" OR "behaviour*" OR "behavior*" OR "language" OR "speech" OR "learning" OR "academic" OR "anxiety" OR "depression" OR "suicid*" OR "health" OR "injur*" OR "illness*" OR "hospital*" OR "health-service*" OR "resilience" OR "strength*"

#4 Title/Abstract/Keyword

"Low-birth-weight" OR "small-for-gestational-age" OR "intrauterine-growth-restrict*" OR "intrauterine-growth-retard*" OR "fetal-growth-restrict*" OR "foetal-growth-restrict*" OR "preterm" OR "pre-term" OR "prematur*"

#5 Title/Abstract/Keyword

"pre-schooler*" OR "preschooler*" OR "kinder" OR "kinders" OR "kindergarten*" OR "kinder-aged" OR "boy" OR "boys" OR "girl" OR "girls" OR "child" OR "children" OR "childhood" OR "pediatric*" OR "paediatric*" OR "school-age*" OR "schoolage*" OR "schoolchild*" OR "schoolgirl*" OR "schoolboy*"

#6 (#1 OR #2) AND #3 AND #4 AND #5

Limits: English language and yr="2000-Current

Medline Search Terms

1. exp indians, north american/ or exp inuits/ or exp oceanic ancestry group/

2. ((Aborigin* or Indigenous) adj1 (Australia* or New-Zealand* or Canada*)).tw,kf,hw.

3. (Anangu* or Arrernte* or Koori* or Goorie* or Tiwi* or Pitjantjatjara* or Luritja* or Warlpiri* or Torres-Strait-Islander* or First-Nation* or Inuit* or Metis* or Eskimo* or Cree? or Iroquois* or Tlingit* or Kainai* or Algonquin* or Anishinaabe* or Dene? or Nakoda* or Syilx* or Maori* or Moriori* or tangata-whenua* or Ngapuhi* or Ngati-Porou* or Ngai-Tahu* or Waikato*).tw,kf.

4. north-american-indian*.tw,kf.

5. 1 or 2 or 3 or 4

6. (Dental or teeth or gum or enamel or body-mass-index or bmi or respiratory or asthma* or disabilit* or vision or eye or sight or eyesight or ophthalmic or heart or cardiac or cardio* or hear or hearing or deaf* or disease* or skin or infection* or development* or milestone? or behavio?r* or language or speech or learning or academic or anxiety or depression or suicid* or health or injur* or illness* or hospital* or health-service? or resilience or strength?).tw,kf,hw.

7. exp Infant, Low Birth Weight/

8. (Low-birth-weight or small-for-gestational-age or intrauterine-growth-restrict* or intrauterine-growth-retard* or f?etal-growth-restrict*).tw,kf.

9. exp infant, premature/ or exp infant, premature, diseases/ or Premature Birth/

10. (preterm or pre-term or prematur*).tw,kf.

11. 7 or 8 or 9 or 10

12. (pre-schooler* or preschooler* or kinder or kinders or kindergarten* or kinder-aged or boy or boys or girl or girls or child or children or childhood or pediatric* or paediatric* or school-age* or schoolage* or schoolchild* or schoolgirl* or schoolboy*).tw,kf,hw,in.

13. 5 and 6 and 11 and 12

14. limit 13 to (case reports or comment or editorial or guideline or letter or practice guideline)

15. 13 not 14

16. limit 15 to (english language and yr="2000 -Current")

Web Of Science Search Terms

#1 **TS**=((Aborigin* OR Indigenous) NEAR/1 (Australia* OR New-Zealand* OR Canada*)) OR **TI**=((Aborigin* OR Indigenous) NEAR/1 (Australia* OR New-Zealand* OR Canada*)) OR **AB**=((Aborigin* OR Indigenous) NEAR/1 (Australia* OR New-Zealand* OR Canada*))

#2 TS=(Anangu* OR Arrernte* OR Koori* OR Goorie* OR Tiwi* OR Pitjantjatjara* OR Luritja* OR Warlpiri* OR Torres-Strait-Islander* OR First-Nation* OR Inuit* OR Metis* OR Eskimo* OR Cree OR Crees OR Iroquois* OR Tlingit* OR Kainai* OR Algonquin* OR Anishinaabe* OR Dene OR Denes OR Nakoda* OR Syilx* OR Maori* OR Moriori* OR tangata-whenua* OR Ngapuhi* OR Ngati-Porou* OR Ngai-Tahu* OR Waikato* OR north-american-indian*) OR TI=(Anangu* OR Arrernte* OR Koori* OR Goorie* OR Tiwi* OR Pitjantjatjara* OR Luritja* OR Warlpiri* OR Torres-Strait-Islander* OR First-Nation* OR Inuit* OR Metis* OR Eskimo* OR Cree OR Crees OR Iroquois* OR Tlingit* OR Kainai* OR Algonquin* OR Anishinaabe* OR Dene OR Denes OR Nakoda* OR Syilx* OR Maori* OR Moriori* OR tangata-whenua* OR Ngapuhi* OR Ngati-Porou* OR Ngai-Tahu* OR Waikato* OR north-american-indian*) OR AB=(Anangu* OR Arrernte* OR Koori* OR Goorie* OR Tiwi* OR Pitjantjatjara* OR Luritja* OR Warlpiri* OR Torres-Strait-Islander* OR First-Nation* OR Inuit* OR Metis* OR Eskimo* OR Cree OR Crees OR Iroquois* OR Tlingit* OR Kainai* OR Algonquin* OR Anishinaabe* OR Dene OR Denes OR Nakoda* OR Syilx* OR Maori* OR Moriori* OR tangata-whenua* OR Ngapuhi* OR Ngati-Porou* OR Ngai-Tahu* OR Waikato* OR north-american-indian*)

#3

TS=(Dental OR teeth OR gum OR enamel OR body-mass-index OR bmi OR respiratory OR asthma* OR disabilit* OR vision OR eye OR sight OR eyesight OR ophthalmic OR heart OR cardiac OR cardio* OR hear OR hearing OR deaf* OR disease* OR skin OR infection* OR development* OR milestone* OR behaviour* OR behavior* OR language OR speech OR learning OR academic OR anxiety OR depression OR suicid* OR health OR injur* OR illness* OR hospital* OR health-service* OR resilience OR strength*) OR TI=(Dental OR teeth OR gum OR enamel OR body-mass-index OR bmi OR respiratory OR asthma* OR disabilit* OR vision OR eye OR sight OR eyesight OR ophthalmic OR heart OR cardiac OR cardio* OR hear OR hearing OR deaf* OR disease* OR skin OR infection* OR development* OR milestone* OR behaviour* OR behavior* OR language OR speech OR learning OR academic OR anxiety OR depression OR suicid* OR health OR injur* OR illness* OR hospital* OR

health-service* OR resilience OR strength*) OR AB=(Dental OR teeth OR gum OR enamel OR body-mass-index OR bmi OR respiratory OR asthma* OR disabilit* OR vision OR eye OR sight OR eyesight OR ophthalmic OR heart OR cardiac OR cardio* OR hear OR hearing OR deaf* OR disease* OR skin OR infection* OR development* OR milestone* OR behaviour* OR behavior* OR language OR speech OR learning OR academic OR anxiety OR depression OR suicid* OR health OR injur* OR illness* OR hospital* OR health-service* OR resilience OR strength*)

#4

TS=(Low-birth-weight OR small-for-gestational-age OR intrauterine-growth-restrict* OR intrauterine-growth-retard* OR fetal-growth-restrict* OR foetal-growth-restrict* OR preterm OR pre-term OR prematur*) OR TI=(Low-birth-weight OR small-for-gestational-age OR intrauterine-growth-restrict* OR intrauterine-growth-retard* OR fetal-growth-restrict* OR foetal-growth-restrict* OR preterm OR pre-term OR prematur*) OR AB=(Low-birth-weight OR small-for-gestational-age OR intrauterine-growth-restrict* OR intrauterine-growth-retard* OR fetal-growth-restrict* OR foetal-growth-restrict* OR preterm OR pre-term OR prematur*)

#5

TS=(pre-schooler* OR preschooler* OR kinder OR kinders OR kindergarten* OR kinder-aged OR boy OR boys OR girl OR girls OR child OR children OR childhood OR pediatric* OR paediatric* OR school-age* OR schoolage* OR schoolchild* OR schoolgirl* OR schoolboy*) OR TI=(pre-schooler* OR preschooler* OR kinder OR kinders OR kindergarten* OR kinder-aged OR boy OR boys OR girl OR girls OR child OR children OR childhood OR pediatric* OR paediatric* OR school-age* OR schoolage* OR schoolchild* OR schoolgirl* OR schoolboy*) OR AB=(pre-schooler* OR preschooler* OR kinder OR kinders OR kindergarten* OR kinder-aged OR boy OR boys OR girl OR girls OR child OR children OR childhood OR pediatric* OR paediatric* OR school-age* OR schoolage* OR schoolchild* OR schoolgirl* OR schoolboy*)

#6 (#1 OR #2) AND #3 AND #4 AND #5

Limits: English language and yr="2000-Current
